# Supplementary material for: Detection and density of breeding marsh birds in Iowa wetlands
Source: PLoS One. 2020 Jan 24;15(1):e0227825. doi: 10.1371/journal.pone.0227825 (PMC6980496; doi:10.1371/journal.pone.0227825)
Supplement: Attachment — Submitted filename: PONE-D-19-26684_Response to Reviewers.docx [file pone.0227825.s002.docx]

**Response to Reviewers**

PONE-D-19-26684

Detection and density of breeding marsh birds in Iowa wetlands

PLOS ONE

**Our responses to comments are in bold text.**

Editor’s comments

1. When submitting your revision, we need you to address these additional requirements.

 Please ensure that your manuscript meets PLOS ONE's style requirements, including those for file naming. The PLOS ONE style templates can be found at

<http://www.journals.plos.org/plosone/s/file?id=wjVg/PLOSOne_formatting_sample_main_body.pdf> and <http://www.journals.plos.org/plosone/s/file?id=ba62/PLOSOne_formatting_sample_title_authors_affiliations.pdf>

2.  In your Methods section, please provide additional location information of the study sites, including geographic coordinates for the data set if available.

**Response: We included a list of the 30 shallow lakes, along with the county and UTM coordinates. These were included as supplementary material (S1 Table).**

3.  In your Methods section, please provide additional information regarding the permits you obtained for the work. Please ensure you have included the full name of the authority that approved the field site access and, if no permits were required, a brief statement explaining why.

**Response: Technically, we are not required to have any permits for this work because we were not handling birds. Our general bird work is authorized under USGS Bird Banding Permit #23285 to Stephen J. Dinsmore. We included a statement at the end of the “Bird surveys” section that states: “Because we were not handling birds, we were not required to have approval by the Iowa State University Institutional Animal Care and Use Committee.”**

4.  We note that you have stated that you will provide repository information for your data at acceptance. Should your manuscript be accepted for publication, we will hold it until you provide the relevant accession numbers or DOIs necessary to access your data. If you wish to make changes to your Data Availability statement, please describe these changes in your cover letter and we will update your Data Availability statement to reflect the information you provide.

**Response: We will deposit our data in Iowa State University’s DataShare if the publication is accepted.**

Reviewer 1 Comments

I thought the paper was well written, informative work that could have some impact for future studies.

**Response: We appreciate this statement.**

The methods section:
Line 110 - you may want to define what palustrine / lacustrine systems are - mentioned several times but I didn't see a definition for the terms. Is it a difference in water regime, wetland size, water depth? Veg community different?

**Response: Thank you for these comments and questions. We defined the terms palustrine and lacustrine in the Study Area section. Based on Cowardin et al. (1979), one of the biggest differences between these is the presence or absence of vegetation. Lacustrine wetlands generally lack vegetation, while palustrine wetlands are characterized by hydrophytes.**

What are the native vegetation communities for the wetlands you studied - what is the goal for restoration? You mentioned restoration processes for the small lakes (removing fish, removing water structures, mimicking natural water regimes) and allowing a 2 year period for vegetation to reestablish - is the type of vegetation important? Could this have had some impact on detection for secretive marsh birds (eg - least bittern have a different habitat preference than sora or viginia rail)? The changes in vegetation community is mentioned several times, but there isn't really any explanation as to what that community is. A brief explanation of what a successful restoration looks like could be helpful here.

**Response: We included more explanation on the vegetation community and the meaning of restoration success in the introduction. Specifically, we further described vegetation characteristics of prairie shallow lakes and how they can be impacted by hydrological fluctuations, invasive fish species, and excessive nutrient loading. We also emphasized the meaning of a successful restoration based on work by other researchers.**

Was distance to roads taken into account when you established your survey points, or was the effect of disturbance from traffic negligible?

**Response: We did not take into account distance to roads. We believe disturbance from roads was negligible because most of the roads near these shallow lakes were either (a) dead-end access roads to the wetland, or (b) rural roads that were not heavily used.**

At line 402 you mention the development of sedge meadow and wet prairie habitat near the edges of the shallow lakes as a positive for the bird communities - are these common for restored shallow lakes in Iowa?

**Response: The presence of sedge meadow and wet prairie habitat was not quantified, as most of our surveys were completed away from shallow lake edges. However, the authors and observers did notice a greater abundance and presence of such habitat while completing these surveys. We clarified this in the discussion.**

Overall - good paper, I think the issues I found were very minor, and it could be published without too many changes.
